# The Impact of Compassion Fatigue on the Psychological Well-Being of Nurses Caring for Patients with Dementia: A Cross-Sectional Post-COVID-19 Data Analysis

**DOI:** 10.3390/healthcare14020224

**Published:** 2026-01-16

**Authors:** Maria Topi, Paraskevi Tsioufi, Evangelos C. Fradelos, Foteini Malli, Evmorfia Koukia, Polyxeni Mangoulia

**Affiliations:** 1 1st Department of Psychiatry, Eginitio Hospital, National and Kapodistrian University of Athens, 115 28 Athens, Greece; 2 “Psychiatriki” Rehabilitation Unit–Pain Management Center, 412 21 Larissa, Greece; 3 Department of Nursing, University of Thessaly, 415 00 Larissa, Greecemallifoteini@yahoo.gr (F.M.); 4 Laboratory Nursing Counselling and Psychoeducation of Patients and Caregivers, Faculty of Nursing, National and Kapodistrian University of Athens, 115 27 Athens, Greece

**Keywords:** nurses, dementia patients, compassion fatigue, quality of life in healthcare, compassion satisfaction, mental health, post pandemic

## Abstract

**Background/Objectives:** Nurses are susceptible to compassion fatigue due to the nature of their professional responsibilities. Factors contributing to this vulnerability include daily patient interactions and organizational elements within their work environment, as well as work-related stress and sociodemographic characteristics, including age, marital status, years of professional experience, and, notably, gender. This research investigates the relationship between compassion fatigue and the levels of anxiety and depression, as well as the professional quality of life among nurses providing care to dementia patients in Greece. **Methods:** A cross-sectional survey was carried out with 115 nurses working in dementia care centers in Greece. The Hospital Anxiety and Depression Scale (HADS), the Professional Quality of Life Scale (ProQOL-5), and the participants’ personal, demographic, and professional information were all included in an electronic questionnaire. Multiple regression analysis was used. **Results:** A total of 42.6% of nurses rated their working environment as favorable. Additionally, 23.5% of the sample exhibited high levels of compassion satisfaction, whereas 46.1% demonstrated low levels of burnout. Female gender (*p* = 0.022) and a higher family income (*p* = 0.046) was positively associated with compassion satisfaction. Regression analysis indicated that elevated symptoms of anxiety and depression were found to correlate with decreased compassion satisfaction, increased burnout, and heightened secondary post-traumatic stress. **Conclusions:** Engaging in the care of patients with dementia, particularly throughout the pandemic period, has underscored a pronounced susceptibility to compassion fatigue, physical fatigue, pain, psychological stress, and a reduced quality of life. These results highlight the importance for nursing management to adopt specific organizational measures, including proper staffing levels, balancing workloads, and conducting routine mental health assessments.

## 1. Introduction

In recent years, increased life expectancy and a growing elderly population have contributed to a rise in the prevalence of chronic conditions, including dementia. According to data from the World Health Organization, over 55 million individuals worldwide are currently living with dementia. Furthermore, dementia is identified as the seventh leading cause of mortality and disability among older adults [1]. Projections indicate that this number will increase to approximately 131.5 million by 2050 [2]. The prevalence of dementia varies across different ethnic groups, as well as according to educational attainment and socioeconomic status. In the United States, higher rates of dementia have been observed among Hispanic and Black populations compared to White populations [3]. Dementia encompasses a spectrum of physical, behavioral, and neuropsychiatric manifestations, some of which pose significant risks to both affected individuals and their caregivers [4]. Hospitalization rates for patients with dementia are reported to be 1.49 times greater than those for patients without dementia, thereby imposing substantial demands on nursing staff in terms of responsibility, commitment, and workload. These challenges frequently elicit adverse emotional responses among nurses, including anxiety, fear, sadness, and frustration [5,6]. Concurrently, the quality of nursing care provided to this patient group appears to have a considerable impact on overall treatment outcomes. Nonetheless, several factors impede the provision of effective nursing care for patients with dementia, notably deficiencies in knowledge, experience, and clinical competence [7].

The concept of compassion fatigue was first introduced by Joinson [8] and later expanded by Figley [9] and Stamm [10]. The earliest reference to this concept in the Greek literature appears in the PhD dissertation of Mangoulia [11]. This phenomenon is often used interchangeably with terms such as secondary traumatic stress, secondary traumatic stress disorder, vicarious stress, and burnout, which has led to some conceptual ambiguity. Fundamentally, compassion fatigue is characterized as a state of exhaustion arising from caregiving relationships and is marked by a diminished capacity to cope [12].

The repercussions of compassion fatigue manifest through a variety of physical and emotional symptoms, including sleep disturbances, impaired concentration, feelings of burden, fear, anxiety, fatigue, emotional collapse, despair, and social isolation, accompanied by detachment. Additional reported effects encompass emotional disconnection, a diminished sense of fulfillment, spiritual emptiness, feelings of incompetence, dissatisfaction, and lack of motivation. Collectively, these symptoms contribute to a decline in the overall quality of professional life among affected individuals [13].

In the nursing profession, several factors have been identified as contributors to compassion fatigue. These include caring for patients with poor prognoses and experiencing patient death [13]. Additional contributing elements pertain to the work environment, such as staff shortages, excessive workloads, unrealistic expectations from patients and their families, emotional involvement with patients, insufficient knowledge and skills, and a lack of support from supervisors and colleagues, which can engender feelings of isolation [14,15].

Effective strategies for managing compassion fatigue include practicing self-care, seeking mutual support among colleagues, and utilizing assistance from social and familial networks. Additionally, maintaining professional boundaries within the workplace, engaging in physical exercise, spirituality, ongoing education, and acquiring information about compassion fatigue have been identified as beneficial. Serving as role models for more experienced colleagues also contributes positively to coping mechanisms [14].

Nursing staff who care for patients with chronic conditions such as dementia experience elevated levels of stress. The literature attributes this to several factors, including the time-intensive nature of care, the behavioral and psychiatric symptoms associated with dementia, the necessity of establishing and maintaining therapeutic relationships despite fears of aggression, and the resultant emotional detachment or apathy toward these patients. Furthermore, nurses often act as intermediaries among family members, patients, and physicians while contending with limited material resources, bureaucratic demands that detract from patient care, and heavy workloads [16,17,18,19].

Numerous studies have demonstrated that disruptions in daily routines—such as decreased physical activity, social isolation, and sleep disturbances caused by confinement—adversely impacted the physical and mental health of older adults during the COVID-19 pandemic [20,21]. The pandemic affected not only the general population but also vulnerable groups with chronic health conditions, including individuals with dementia and their caregivers.

Cognitive impairments in dementia patients have hindered adherence to infection control measures, thereby increasing their risk of infection and mortality [20,21]. In many cases, protective protocols in dementia care facilities were particularly stringent to safeguard this vulnerable population. However, these measures resulted in significant adverse effects, including psychomotor agitation, sleep disturbances, and depressive symptoms. Comparable psychological and physical stress has been reported among nurses caring for dementia patients [22,23,24,25]. A study conducted in Switzerland found that nurses working with dementia patients primarily experienced emotions of fear, helplessness, anxiety, and loneliness during the pandemic. Nonetheless, these nurses also reported enhanced levels of cooperation and job satisfaction, which were attributed to the formation of family-like relationships during periods of isolation and the strengthening of collegial bonds [26,27,28].

The COVID-19 pandemic placed a significant psychosocial strain on nurses worldwide, with consistently high rates of anxiety, depression, and post-traumatic stress reported. Global data showed that about 37% of nurses experienced depressive symptoms, while over 40% suffered from anxiety [29]. Similar patterns were observed in national studies: in Italy, 22% of dementia care staff reported anxiety and 39% PTSD [30], whereas in Spain, healthcare workers faced even higher rates of depression (49%), anxiety (59%), and PTSD (71%) [31]. In Germany, more than half of nurses reported considerable psychological distress, including moderate to severe levels of stress, anxiety, and depression [32,33].

In addition to mood and anxiety disorders, nurses faced increased workloads, burnout, compassion fatigue, and greater physical and mental strain during the pandemic [34,35]. These difficulties were especially severe in dementia care settings, where infection control protocols, social isolation, and the complex behavioral and emotional needs of patients heightened occupational stress and fatigue [36].

Psychosocial vulnerability among nurses was shaped by both personal and organizational factors such as gender, age, nursing specialty, direct contact with COVID-19 patients, and involvement in dementia care [32,33]. Structural issues—including staff shortages, limited resources, unclear care guidelines, and inadequate psychological support—further intensified stress and burnout among dementia care nurses [37,38,39]. Moreover, the unique challenges of dementia care, like patients’ poor compliance with protective measures, behavioral symptoms, and ethical decision-making responsibilities, contributed to ongoing mental health difficulties for nursing staff [20,40,41].

Both prior to and during the pandemic, nursing personnel in dementia care settings reported considerable stress, emotional exhaustion, and frustration, with these indicators intensifying markedly throughout the pandemic [42,43]. Additionally, nursing staff indicated a lack of adequate training prior to the pandemic to effectively manage emergency situations and address the specific needs of this patient population [44].

Protective factors that mitigated psychosocial burden included workplace support, clear and consistent guidelines and protocols related to the virus, involvement in decision-making processes, and enhanced communication among colleagues [33]. Given the particularly demanding nature of dementia care, providing frequent, even brief, breaks during shifts was identified as especially beneficial and supportive for nursing staff [45].

While compassion fatigue among nurses has been extensively studied, there is a notable paucity of research specifically addressing nurses who care for patients with dementia in Greece, especially in the post-COVID-19 context. Previous Greek studies have primarily focused on general hospital populations [15,30,46], limiting the understanding of the unique emotional and psychological challenges associated with dementia care. Moreover, there is a significant lack of post-pandemic research examining the combined effects of compassion fatigue, burnout, and secondary traumatic stress on nurses’ mental health outcomes, including anxiety and depression.

In response to these research gaps, the present study constitutes one of the first post-pandemic examinations of professional quality of life and psychological well-being among dementia care nurses in Greece. By analyzing the interrelations among compassion fatigue, burnout, secondary traumatic stress, anxiety, and depression through the application of validated psychometric tools, this research provides a contextually grounded contribution that builds upon international findings from Italy, Spain, and Germany [30,31,32]. Consequently, it offers novel empirical evidence and informs culturally and organizationally tailored interventions designed to support nurses working in dementia care settings.

To clarify measurement consistency, the present study defines compassion fatigue as encompassing two components—burnout and secondary traumatic stress—measured using the ProQOL-5 scale.

Furthermore, the term “post-COVID-19” denotes the period following the complete removal of all national restrictions in Greece at the beginning of 2023.

The objective of this study is to examine the relationship between compassion fatigue and nurses who provide care for patients with dementia. Specifically, the study aims to assess the impact of compassion fatigue on the mental health of these nurses. The research questions guiding this investigation are as follows:

- What is the level of compassion fatigue among nurses caring for patients with dementia?
- What is the status of mental health, specifically anxiety and depression, among nurses caring for patients with dementia?
- Which demographic, individual, and professional characteristics of nurses are associated with compassion fatigue and mental health outcomes in those caring for patients with dementia?
- What is the relationship between compassion fatigue and the psychological well-being of nurses providing care to patients with dementia?

## 2. Materials and Methods

### 2.1. Data Collection

This research employs a non-experimental, cross-sectional survey and correlational design utilizing primary quantitative data. The quantitative methodology was selected due to its capacity to objectively capture respondents’ responses. Subsequently, the collected data were transformed into numerical and statistical formats, enabling the execution of suitable comparative analyses between variables to derive the study’s findings.

### 2.2. Participants

The present study was conducted exclusively among nurses working in four nursing units dedicated to the care of dementia patients within the prefecture of Larissa. The study population, which constituted the final sample, comprised 115 nurses possessing a minimum of one year of prior professional experience, regardless of gender or age, and with the ability to read and comprehend Greek. Of the 130 nurses invited, 115 completed the questionnaire (response rate: 88.5%).

Participants were recruited using a convenience sampling technique, which involves selecting the most readily accessible and available individuals who consented to participate in the survey. This approach facilitated the acquisition of a substantial sample size within a relatively brief timeframe.

Data collection occurred between early February 2024 and the end of March 2024, representing a post-pandemic stabilization period in healthcare practice. Questionnaires were distributed and collected via the Google Forms platform, with an estimated completion time of approximately 20 min per respondent. Prior to participation, all respondents received written information detailing the study’s objectives, the voluntary nature of their involvement, and assurances of the anonymity of their responses.

### 2.3. Research Instrument

Three validated instruments were used to collect data:

Section 1: Demographic and Professional Characteristics—This section gathered participants’ personal and occupational information (18 items) such as gender, age, marital status, number of children, educational attainment, years of service, job title, working hours, night shifts, monthly income, and job satisfaction.

Section 2: Professional Quality of Life Scale (ProQOL, Version 5)—This tool assesses Compassion Satisfaction, Burnout, and Secondary Traumatic Stress. It includes 30 items (10 per subscale) on a 5-point Likert scale (0 = Never to 5 = Always). The Greek adaptation by Missouridou et al. [38,39] reported Cronbach’s α ranging from 0.80 to 0.89.

Section 3: Hospital Anxiety and Depression Scale (HADS)—A 14-item self-report instrument measuring anxiety (7 items) and depression (7 items). Each item is scored 0–3, with total subscale scores ranging 0–21. The Greek version shows high internal consistency (α = 0.887).

This study employed a questionnaire comprising three distinct instruments for data collection. The first section addressed the respondents’ personal, demographic, and professional characteristics. This section included eighteen items covering variables such as gender, age, marital status, number of children, educational attainment, years of service, job title, leadership responsibilities, working hours, frequency of night shifts, number of weekends worked per month, patient load within the department, monthly family income, work environment, job satisfaction within the nursing profession, motivations for choosing the profession, methods of relaxation after work, and whether any family member has experienced dementia or Alzheimer’s disease.

The second section focused on assessing nurses’ quality of life through the Professional Quality of Life Scale (ProQOL-5), specifically the Compassion Satisfaction and Compassion Fatigue Version 5 [47]. This instrument evaluates the risk of compassion fatigue and burnout—negative outcomes associated with caregiving professions—as well as the level of compassion satisfaction experienced by the individual. Each of the three dimensions is measured via ten items, rated on a five-point Likert scale ranging from 0 (Never) to 5 (Always) [48,49].

Given the direct interpersonal nature of caregiving, the compassion provided can have both beneficial and adverse effects on caregivers. Participants were instructed to reflect on their experiences over the preceding 30 days and to respond candidly to items concerning both positive and negative aspects of their caregiving roles.

Cultural adaptation and validation of the instrument were conducted by Missouridou et al. [50,51].

Part 3 focused on the Hospital Anxiety and Depression Scale (HADS), a brief 14-item self-report instrument designed to evaluate symptoms of anxiety and depression, particularly in individuals with coexisting physical health conditions. The HADS is endorsed by European consensus guidelines for caregivers of individuals with dementia and has been employed as a primary outcome measure in extensive clinical trials within this population [52].

Analyzing the factor structure of the HADS among caregivers also facilitates the assessment of measurement invariance, which is crucial for determining whether the scale can reliably detect differences across caregiver subgroups. This consideration is particularly important given the heterogeneity of caregiver populations and the research interest in how specific caregiver characteristics—such as gender, kinship relation, cohabitation status with the person with dementia, or age—may influence levels of stress or depression [52].

The HADS evaluates three dimensions: depression, anxiety, and negative emotionality. Originally developed in 1983 by A. S. Zigmond and R. P. Snaith, the HADS-7 is a self-administered scale intended to screen for anxiety and depression [52]. While the HADS-14 does not provide a definitive clinical diagnosis, it serves as an initial screening tool within a multi-stage assessment process, identifying individuals at elevated risk who warrant further clinical evaluation.

The scale assesses participants’ emotional states by asking them to indicate the extent to which they have experienced specific feelings over the preceding week. It comprises seven items related to anxiety and seven related to depression, each rated on a four-point Likert scale ranging from 0 (Not at all) to 3 (All the time/often) [52].

Scores for each subscale are calculated by summing the responses to the respective items, yielding a range from 0 to 21. Scores between 0 and 7 are considered within the normal range, indicating non-pathological levels of anxiety or depression. Scores from 8 to 10 suggest borderline or intermediate conditions, representing doubtful cases, while scores from 11 to 21 denote clinically significant symptoms warranting appropriate intervention. These thresholds apply equally to both the Anxiety and Depression subscales [52].

The HADS has been adapted and translated into Greek through a process involving independent back-translation. The Greek version demonstrates high internal consistency, with a Cronbach’s alpha coefficient of 0.887.

All measurement instruments were translated and psychometrically validated for use within the Greek population. Specifically, the Professional Quality of Life Scale version 5 (ProQOL-5) was translated and culturally adapted by Missouridou et al. [50,51]. In the present sample, the internal consistency reliability coefficients (Cronbach’s alpha) were as follows: Compassion Satisfaction, α = 0.89; Burnout, α = 0.82; and Secondary Traumatic Stress, α = 0.84. Additionally, the Greek version of the Hospital Anxiety and Depression Scale (HADS), as validated by Mystakidou et al. [52], exhibited a Cronbach’s alpha of 0.887.

### 2.4. Ethical Considerations

This research was conducted following the approval of the questionnaire by the Ethics and Deontology Committee of the University of Thessaly. Additionally, authorization was obtained individually from the respective scientific councils of each healthcare facility where the questionnaires were administered.

Participants were assured of the anonymity and confidentiality of their personal information, and no financial burden was imposed on the healthcare institutions. Prior to participation, all individuals were provided with an information and consent form, which detailed that their responses would be treated with strict confidentiality and anonymity, thereby safeguarding their personal data.

Furthermore, permission was obtained from the original authors for the use of all measurement scales incorporated in the questionnaire.

### 2.5. Statistical Analysis

The Kolmogorov-Smirnov test was used to assess the normality of the distributions of quantitative variables. For quantitative variables, descriptive statistics were presented as means and standard deviations (SDs) or medians and interquartile ranges (IQRs). Qualitative variables were summarized using absolute (N) and relative frequencies (%). In questions with the option of selecting more than one answer (e.g. methods for relaxation), the percentage was calculated by dividing the absolute frequency with the total sample size. The correlation between professional quality of life (ProQOL-5) and depression/anxiety (HADS) scales was evaluated using Spearman’s correlation coefficients (rho). Multiple linear regression, in an enter method, was used to identify independent predictors of ProQOL and HADS scales. Demographics and work-related characteristics were entered as independent variables. When HADS scales were the dependent variables, ProQOL-5 scales were entered as independent variables in the analysis, in a stepwise method (*p* = 0.05 for entry and *p* = 0.010 for removal), due to their high intercorrelation; thus, only the significant ProQOL-5 scales remained in the models. Since there was no normal distribution in HADS subscales, linear regression with them as dependent variables was conducted after having them logarithmically transformed. From the regression analyses, unstandardized regression coefficients (Betas), Standard errors (SEs), Standardized regression coefficients (Standardized betas), 95% confidence interval of Betas (95% CI), Multicollinearity diagnostic values (such as VIF, Tolerance) and adjusted Coefficient of Determination (adjusted R^2^) emerged. Missing values were treated in analysis through case-wise deletion, since there were only a few (3 at most). Due to low missing values, comparisons between responders and non-responders were not conducted. Comon-methods variance bias was tested with Harman’s test and it was found that there was not an issue (variance < 50%). All statistical tests were two-tailed, with significance set at 5%. It was calculated that with the sample size of 115 participants, the study would have 95% power to perform multiple linear regression analysis with dependent variables as the study outcomes, at a significance level of 0.05 and for effect sizes equal or greater than 0.25. Statistical analyses were performed using the SPSS software, version 26.0.

## 3. Results

### 3.1. Demographic Characteristics of Participants

The study sample comprised 115 healthcare professionals, with a mean age of 37.7 years (SD = 10.6). Females constituted 75.7% of the participants. Regarding marital status, the majority (44.3%) were married. In terms of parental status, the majority (46.1%) reported having no children. Educationally, the majority (46.1%) were graduates of Technical Vocational High Schools specializing in Nursing. Additionally, 4.3% held university degrees, 1.7% possessed a master’s degree, while none had attained a doctoral degree. The mean monthly income among participants was 1128.3 € (SD = 518.6 €). A comprehensive summary of the participants’ demographic characteristics is presented in Table 1.

Table 2 presents the working characteristics of the participants, who exhibited a median tenure of 5 years, ranging from 3 to 14 years. The majority of participants (76.5%) were nursing assistants with secondary education, followed by 13% who were nurses possessing technical education. Auxiliary staff with compulsory education comprised 6.1%, while nurses with primary education accounted for 4.3%. A minority of 11.3% held positions of responsibility, and 74.8% were engaged in rotating shift work. The median number of night shifts per month was 5, with a range of 3 to 6, and the median number of weekend shifts was 2, ranging from 2 to 3. The median patient census in the department was 50, with a range between 40 and 50. Additionally, when queried about having a family member affected by dementia or Alzheimer’s disease, 13.9% of participants responded affirmatively.

Table 3 presents the professional environments in which the participating nurses were employed. A total of 42.6% of respondents characterized the working atmosphere with their colleagues as good, 25.2% as neutral, while 19.1% and 1.7% described it as very good and very bad, respectively. Regarding job satisfaction, 52.2% reported being quite satisfied with the nursing profession, 20% very satisfied, and 15.7% somewhat satisfied. Motivations for choosing this profession included a desire to help others (39.1%), the increased likelihood of professional rehabilitation (27.0%), and environmental influences (27.0%), whereas 15.7% indicated that they entered the profession by chance. For relaxation, 41.7% preferred sleep, 34.8% socializing with friends, and 27.0% engaging in physical exercise.

### 3.2. Professional Quality of Life Scale (ProQOL-5)

Table 4 presents descriptive statistics for the Professional Quality of Life (ProQOL-5) scale, which comprises three subscales: Compassion Satisfaction, Burnout, and Secondary Traumatic Stress. Elevated scores within each subscale indicate greater levels of compassion satisfaction, burnout, and secondary traumatic stress, respectively. In the current sample, scores on the Compassion Satisfaction subscale documented a mean of 35.4 (SD = 7.4), reflecting moderate to high levels of satisfaction. Scores on the Secondary Traumatic Stress subscale documented a mean of 23.5 (SD = 6.4). The Burnout subscale scores documented a mean of 21.5 (SD = 5.8). Specifically, 23.5% of participants exhibited high levels of compassion satisfaction, 69.6% demonstrated moderate levels, and 7% reported low levels. Regarding professional burnout, 46.1% of participants experienced low levels, while 53.9% exhibited moderate levels; notably, no participants were identified with high levels of professional burnout. In terms of secondary traumatic stress, 58.3% of participants reported low levels, and 41.7% reported moderate levels, with no cases of high secondary traumatic stress observed.

### 3.3. Hospital Anxiety and Depression Scale (HADS)

Table 5 follows, with descriptive data for the anxiety and depression dimensions of the HADS hospital scale. The scores on the dimensions range from 0 to 21 points. A higher score indicates greater anxiety or depression, respectively. In this sample, the median number of depression dimensions was 4, with a range of 2 to 6, and the median number of anxiety dimensions was 3, with a range of 1 to 4.

### 3.4. Scale Correlations

Table 6 presents the Spearman correlation coefficients between the Professional Quality of Life (ProQOL-5) scale and the subscales of the Hospital Anxiety and Depression Scale (HADS). All correlations between the ProQOL-5 dimensions and the HADS subscales were statistically significant. Higher burnout and secondary traumatic stress correlated with higher anxiety and depression (rho ranged from 0.36 to 0.44; *p* < 0.001), while higher compassion satisfaction correlated negatively with these symptoms (rho = −0.37; *p* < 0.001 with depression and rho = −0.22; *p* = 0.016 with anxiety).

### 3.5. Multiple Linear Regressions

To determine the factors independently associated with the dimensions of professional quality of life, anxiety and depression, multiple linear regression analyses were conducted. The dependent variables comprised the scores on these scales, while the independent variables included participants’ demographic and occupational characteristics.

In Table 7, the results of multiple regression analyses with the dimensions of professional quality of life as dependent variables are presented. The model significantly predicted Compassion Satisfaction (F = 5.6, *p* < 0.001), accounting for 37% of the variance (R_adj_^2^ = 0.37). Gender, monthly family income, work climate, and job satisfaction emerged as independent predictors of compassion satisfaction. Specifically, female gender was positively associated with compassion satisfaction (beta = 3.20, t = 2.32, *p* = 0.022, 95% CI [0.47, 5.94]). Additionally, higher family income (*p* = 0.046, 95%) and a more positive perception of the collegial work environment (*p* < 0.001, 95%) were both positively correlated with increased compassion satisfaction. Similarly, greater job satisfaction (*p* = 0.018, 95%) was significantly associated with elevated compassion satisfaction.

Professional burnout was significantly predicted by the model (F = 5.0, *p* < 0.001), which accounted for 32% of the variance (R_adj_^2^ = 0.32). Regarding professional burnout, satisfaction with the work environment and job satisfaction were identified as independent correlates. Notably, higher satisfaction with colleagues corresponded to lower levels of burnout (*p* < 0.001, 95%). Likewise, increased job satisfaction was linked to fewer burnout symptoms (*p* = 0.049, 95%).

The model significantly predicted Secondary Post-traumatic Stress (F = 2.7, *p* = 0.004), accounting for 17% of the variance (R_adj_^2^ = 0.17). Satisfaction with the work environment and educational attainment were independently related to secondary post-traumatic stress. Greater satisfaction with colleagues was associated with lower secondary post-traumatic stress (*p* = 0.007, 95%). Furthermore, vocational Training Institute graduates were linked to lower post-traumatic stress (*p* = 0.015, 95%).

Tolerance values were above 0.10 and VIF under 10; thus, there was no multicollinearity present.

In Table 8, the results of multiple regression analyses with anxiety and depression as dependent variables are presented. The model significantly predicted Anxiety (F = 3.4, *p* < 0.001), accounting for 24% of the variance (R_adj_^2^ = 0.24). Both the interpersonal climate among colleagues and burnout emerged as independent predictors of the anxiety dimension. Specifically, a more positive collegial atmosphere was linked to lower levels of anxiety (*p* = 0.044, 95%), whereas elevated burnout was associated with heightened anxiety (*p* < 0.001, 95%). Depression was significantly predicted by the model (F = 4.0, *p* < 0.001), which accounted for 28% of the variance (R_adj_^2^ = 0.28). Burnout was identified as the sole independent predictor significantly associated with the depression dimension, with higher levels of burnout corresponding to increased severity of depressive symptoms (*p* < 0.001, 95%). Tolerance values were above 0.10 and VIF under 10; thus, there was no multicollinearity present.

## 4. Discussion

### 4.1. Model Validation and Interpretation of Findings

This study investigated the burden experienced by nurses providing care to patients with dementia. Existing research consistently demonstrates that caring for individuals with chronic conditions exerts a substantial impact on nurses’ physical and psychological well-being.

The multiple linear regression analysis employed to evaluate the research hypotheses yielded several notable statistical outcomes. The model significantly predicted compassion fatigue (*p* < 0.001). Within the demographic variables, gender, monthly family income, work climate, and job satisfaction were identified as independent predictors of compassion satisfaction. Professional burnout was also significantly predicted by the model (*p* < 0.001), with satisfaction regarding the work environment and job satisfaction emerging as independent correlations. Furthermore, secondary post-traumatic stress was significantly predicted by the model (*p* = 0.004), with satisfaction with the work environment and educational attainment independently associated with this outcome.

Anxiety was significantly accounted for by the model (*p* < 0.001), with interpersonal climate among colleagues and burnout identified as independent predictors. Finally, the depression variable was significantly predicted by the model (*p* < 0.001), with burnout serving as the sole independent predictor significantly linked to the depression dimension.

### 4.2. Prevalence and Intensity of Compassion Fatigue in Nursing Professionals Caring for Patients with Dementia

In the present sample, scores on the Compassion Satisfaction subscale indicated predominantly moderate to high levels of satisfaction, accounting for 69.6% of participants. Regarding Secondary Traumatic Stress, the data revealed low levels in 58.3% of the sample and moderate levels in 41.7%. For the Burnout subscale, participants exhibited low levels in 46.1% and moderate levels in 53.9% of cases.

In contrast to several studies that documented elevated levels of burnout and compassion fatigue in the immediate aftermath of the pandemic [53,54,55,56], the nurses participating in our study exhibited moderate levels of burnout. This discrepancy may be indicative of a progressive adjustment to post-pandemic work environments and the enhancement of peer support mechanisms. A substantial body of research indicates that the pandemic subjects healthcare professionals to a range of stressors beyond biological risks, including occupational, social, and other factors, thereby positioning occupational stress as a significant public health concern. Additionally, multiple studies conducted prior to the onset of the COVID-19 pandemic have documented that healthcare workers are susceptible to elevated stress levels, which in turn are associated with diminished professional quality of life (ProQoL-5) [55]. Nonetheless, the ongoing presence of anxiety and depressive symptoms implies that emotional distress remains a significant concern within this population.

### 4.3. Psychological Well-Being and Mental Health Status, Including Anxiety and Depression, Among Nurses Providing Care for Patients with Dementia

The overall levels of anxiety and depression observed within the study sample were relatively low. Pre-pandemic data indicated that approximately one to two nurses reported experiencing work-related stress that adversely affected their well-being, with mental health nurses being particularly affected. Notably, a substantial proportion of nurses experience symptoms of anxiety and depression, with prevalence rates exceeding those observed in the general population [57,58]. Furthermore, exposure to traumatic and distressing events in the workplace renders nurses susceptible to post-traumatic stress disorder and an elevated risk of suicide. Work-related mental health issues have significant implications for staff retention, sickness absenteeism, presenteeism, and the quality of patient care [59]. Consequently, these concerns should be acknowledged as critical occupational health and safety risks.

Data collected during the COVID-19 pandemic indicated that mental health nurses exhibited lower levels of anxiety and experienced moderate levels of COVID-19-related burnout, with family support appearing to serve as a protective factor [60]. Additionally, mental well-being was found to be associated with nurses’ educational attainment. Advanced practice nurses, those with postgraduate qualifications, and those employed in outpatient settings generally reported higher levels of mental wellness compared to their counterparts, potentially attributable to a reduced workload associated with their higher professional status [61]. Furthermore, during the pandemic, one in two healthcare workers reported symptoms of depression or anxiety, underscoring the critical importance of promptly identifying and addressing mental health symptoms among healthcare professionals in the context of COVID-19 [62].

Post-pandemic research has revealed elevated levels of anxiety and depression among nurses, as well as healthcare workers more broadly. These prevalence rates were comparable to those observed in the general population [63,64], potentially reflecting the pandemic’s widespread impact as a psychological shock affecting overall mental well-being. The occurrence of post-traumatic stress disorder (PTSD) and acute stress symptoms among healthcare workers may be attributed to their frequent exposure to numerous sudden and severe fatalities. Repeated or intense exposure to distressing details of traumatic events is recognized as a significant risk factor for trauma. Within the emergency context of the COVID-19 pandemic, healthcare professionals encountered multiple potentially traumatic stressors. Additionally, the absence of adequate social support emerged as a critical detrimental factor for the mental health of healthcare workers, exacerbated by quarantine measures, perceived stigmatization, fears of transmitting the virus to family members, unpredictability in daily patient caseloads, the necessity to manage patient and family expectations under unforeseen circumstances, the burden of complex decision-making, elevated daily mortality rates, and continual modifications to hospital protocols [64,65,66].

### 4.4. Demographic, Personal, and Occupational Determinants Related to Compassion Fatigue and Psychological Well-Being in Nurses Providing Care to Patients with Dementia

The findings of this study indicate that a positive collegial environment and elevated job satisfaction are correlated with lower levels of burnout and secondary traumatic stress. These results are consistent with prior research demonstrating that supportive professional relationships, effective communication, and supervisory recognition contribute to increased resilience and overall well-being among nurses [67,68,69]. As noted by these scholars, teamwork and a sense of belonging serve as protective factors that mitigate compassion fatigue and burnout. Similarly, the relationship between burnout and mental health observed in our sample aligns with international evidence. More than half of the nurses in the current study reported considerable satisfaction with the nursing profession. Notably, no participants exhibited high levels of burnout, and only 7% reported low job satisfaction. Given that job dissatisfaction is a significant predictor of burnout, maintaining high levels of job satisfaction is essential for preventing burnout and fatigue among nurses and other healthcare professionals [70]. These findings align with those of Coetzee and Klopper, whose research on nurses caring for anemic patients yielded similar results [46]. Professional burnout has been linked to factors such as staffing adequacy, management effectiveness, and the psychological burden inherent in nursing, which involves care provision, pain management, illness, and loss [70,71,72].

Our post-pandemic findings confirm that, although some emotional recovery has occurred, a significant residual psychological burden persists significantly among nurses caring for dementia patients. A significant correlation was identified between professional quality of life and the mental health status of these nurses. Specifically, increased levels of stress and depression reported by participants were inversely related to their overall job satisfaction. Comparable findings have been documented in a study involving nurses caring for dementia patients, which observed a decline in self-esteem that subsequently led to heightened anxiety and depression [73]. Furthermore, research by Finzi-Dottan and Kormosh highlights that compassion fatigue stemming from the challenges of managing complex demands within an overburdened healthcare system places considerable stress on nurses responsible for service provision [16].

Participants in the study reported that their preferred activities for relaxation included sleep (43.2%), socializing with friends (36%), and physical activity or exercise (27.9%). For healthcare professionals specifically, engaging in recreational activities, social interaction, and rest is critically important for maintaining mental resilience [74]. However, the ability to participate in these activities was often constrained by movement restrictions imposed during the pandemic, thereby imposing a substantial burden on healthcare workers.

Workplace atmosphere and burnout were independently correlated with stress levels; a more positive collegial environment was linked to lower stress, while higher burnout corresponded with increased stress. The literature suggests that mentorship and guidance from more experienced colleagues serve as effective strategies for mitigating depression, stress, and burnout among nurses [14,75]. Nurses employed in dementia care units in the United States reported pervasive stress, frustration, and burnout, largely attributable to diminished support from management and peers, as well as understaffing [44]. These findings align with pre-pandemic research [76,77].

Gender, monthly family income, work environment, and job satisfaction were identified as independent predictors of compassion satisfaction. Female gender was positively associated with compassion satisfaction, with female participants exhibiting higher levels of compassion satisfaction compared to their male counterparts. Additionally, increased family income correlated positively with greater compassion satisfaction. A more favorable perception of the collegial work atmosphere was also linked to enhanced compassion satisfaction. Similarly, higher overall job satisfaction corresponded with increased compassion satisfaction.

Both satisfaction with the work environment and job satisfaction were independently linked to professional burnout. Specifically, greater satisfaction with colleagues was inversely related to burnout levels. Similarly, higher job satisfaction was associated with lower burnout. The presence of supportive peers and colleagues has been shown to mitigate stress and enhance compassion satisfaction [14,75].

Satisfaction with the work environment and educational attainment were independently associated with secondary post-traumatic stress. Higher satisfaction with the collegial work environment corresponded to lower levels of secondary post-traumatic stress. Graduates of Vocational Training Institutes were linked to lower post-traumatic stress. Furthermore, these graduates reported less secondary post-traumatic stress compared to those who graduated from the Technical Vocational High School (T.E.L.) Nursing Department. Engagement in training opportunities and participation in nursing workshops positively influenced compassion fatigue and stress outcomes [14,75]. A longitudinal study conducted three years following the initial wave of the COVID-19 pandemic revealed a decline in rates of compassion fatigue and burnout among healthcare professionals compared to earlier pandemic phases. Notably, nurses exhibited higher incidences of compassion fatigue, secondary traumatic stress, and professional burnout compared to findings from previous research [78].

### 4.5. Relationship Between Compassion Fatigue and Psychological Well-Being in Nursing Personnel Caring for Patients with Dementia

Notably, compassion satisfaction exhibited a negative correlation with both anxiety and depression, indicating that higher levels of anxiety and more severe depressive symptoms were associated with lower compassion satisfaction. Conversely, the dimensions of burnout and secondary traumatic stress demonstrated positive correlations with anxiety and depression, suggesting that increased burnout and secondary traumatic stress were linked to elevated anxiety and depressive symptoms.

Elevated levels of professional burnout and secondary post-traumatic stress were associated with increased anxiety and depression among nurses in this study. The repercussions of professional burnout on employees’ physical and mental health have significant implications both within and beyond the workplace [79]. Notably, even prior to the COVID-19 pandemic, burnout among healthcare professionals, with attendant effects on mental health, was recognized as a widespread issue [80]. During the pandemic, numerous studies involving healthcare workers reported heightened incidences of mental distress and burnout, partly attributed to social isolation measures and their consequences [81,82]. The pandemic further intensified anxiety related to viral transmission, which correlated with elevated professional burnout, as demonstrated in a study of nurses in India during the initial wave in 2020 [83].

The present study, conducted post-pandemic, establishes a link between substantial levels of professional burnout and increased prevalence of anxiety and depression. Consistent with these findings, a related study involving 92 nurses demonstrated a significant association between work-related stress, depression, emotional exhaustion, and professional burnout. Both emotional and physical exhaustion among nurses were found to be connected to the pandemic context [84]. Consequently, it appears that the pandemic has markedly influenced the incidence of professional burnout as well as elevated rates of anxiety and depression among nursing professionals.

Research focusing on nurses caring for individuals with dementia during the pandemic revealed increased experiences of frustration and emotional exhaustion [44]. Even prior to the pandemic, this group exhibited elevated levels of emotional exhaustion, professional burnout, and depersonalization [42,43]. The demanding nature of dementia care, particularly the challenges inherent in communicating with cognitively impaired patients, exacerbated the pressure on nurses to enforce COVID-19 containment measures among this high-risk population. The absence of adequate training for managing these circumstances further adversely affected nurses’ job satisfaction [85]. Additionally, patient aggression, noncompliance with protective measures, and the imperative to safeguard patients, healthcare workers, and their families collectively contributed to a highly stressful work environment for nurses [44,86].

In summary, our study contributes new evidence from a Greek post-pandemic context, confirming that compassion fatigue and psychological distress among dementia care nurses are influenced not only by individual factors but also by organizational culture. Similar to other international findings, supportive work environments and professional development opportunities serve as key protective factors, whereas limited resources and emotional strain increase vulnerability to burnout.

### 4.6. Limitations of This Study

This study is subject to several limitations. Firstly, the specific temporal and geographical scope of this research may have constrained the diversity of the sample. The limited time frame potentially restricts participant involvement, while the geographical focus poses challenges for the generalizability of the findings, given that many variables under investigation may vary between the capital city and provincial regions. A notable limitation of this study is the small, or relatively limited, sample size, which may constrain the generalizability of the findings. It is acknowledged that this study’s cross-sectional design limits the ability to draw causal conclusions, and it is therefore recommended that future research employ longitudinal methodologies.

Secondly, the assessment instruments employed were self-administered, which introduces a considerable risk of response bias and consequently undermines the validity of the results. Furthermore, the use of a convenience sampling method may compromise the reliability of the findings, as this approach heightens the likelihood of selection bias; individuals who opt to participate voluntarily may differ systematically in their perceptions of work-life quality or mental health from those who decline participation.

Scale Reliability: It is recognized that although the assessment instruments exhibited acceptable reliability, their psychometric properties may vary within this population, potentially leading to measurement bias. Lack of Qualitative Data: The inclusion of qualitative methodologies, such as interviews or focus groups, is considered to have the potential to enhance the depth of contextual insight.

Notwithstanding these limitations, this study’s findings offer valuable insights into the impact of compassion fatigue on the mental health of nurses caring for patients with dementia.

### 4.7. Recommendations for Clinical Practice

Based on the findings, healthcare institutions—both management and staff—should be made aware of the importance of improving the stressful and demanding work environment to the greatest extent possible, promoting self-actualization, personal and professional well-being, mental resilience, the effectiveness and efficiency of nurses, and, by extension, the quality of care provided to patients.

The above findings, combined with the experiences of participating nurses and healthcare professionals in this and similar studies, show that the work environment and support from administrative and supervisory authorities play a significant role in reducing burnout and stress, particularly during times like the COVID-19 pandemic.

## 5. Conclusions

This investigation represents one of the initial comprehensive analyses of compassion fatigue and psychological well-being among nurses providing care to patients with dementia within the post-COVID-19 Greek healthcare milieu, utilizing validated Greek adaptations of established psychometric tools. The results indicate that Greek nurses engaged in dementia care experience moderate levels of compassion fatigue and occupational burnout, alongside low to moderate manifestations of anxiety and depression. These findings corroborate that the psychological burdens documented internationally are equally pertinent within the Greek clinical context.

Significantly, this study identifies several demographic and professional variables linked to nurses’ well-being in Greece. Female gender, advanced age, engagement in shift work, and elevated occupational burnout correlate with increased depressive symptoms and diminished physical health. Conversely, a supportive work environment, higher job satisfaction, and stable family income are associated with reduced psychological distress. Additionally, shift work and lower educational attainment emerge as salient risk factors for heightened stress and physical discomfort. These outcomes underscore structural and organizational vulnerabilities within Greek dementia care services that may predispose nurses to increased psychological strain.

While the cross-sectional and correlational nature of this study limits causal inference, the observed associations offer valuable insights into the multifaceted determinants of nurse well-being in dementia care. From a practical standpoint, the findings emphasize the pressing need for organizational interventions within Greek healthcare institutions aimed at enhancing workplace support, optimizing staffing and scheduling practices, and expanding access to mental health resources. Interventions such as workload management, targeted psychological support, counseling services, and mindfulness-based stress reduction programs may be instrumental in alleviating compassion fatigue and fostering sustainable nursing practices in dementia care settings.

In summary, this study contributes significant localized evidence to the global literature by elucidating the psychological challenges encountered by Greek nurses caring for individuals with dementia and by identifying modifiable occupational factors that may guide future policy and clinical initiatives. Further research employing longitudinal and interventional methodologies is warranted to elucidate causal mechanisms and to develop evidence-based strategies designed to protect the mental health and professional quality of life of nurses in dementia care.

## Figures and Tables

**Table 1:** Demographic characteristics of participating nurses (N = 115).

		N %	
Gender	Male	28	24.3
	Female	87	75.7
Marital status	Unmarried	50	43.5
	Married	51	44.3
	Widowed	1	0.9
	Divorced	9	7.8
	Cohabiting with partner	4	3.5
Number of children	0	53	46.1
	1	23	20.0
	2	30	26.1
	3	9	7.8
Educational attainment	Technical Vocational High School Nursing Department	53	46.1
	Vocational Training Institute Nursing	38	33.0
	Technological Educational Institute	17	14.8
	University	5	4.3
	Master’s Degree	2	1.7
	Doctorate	0	0.0
		Mean (SD)	
Age (years)		37.7 (10.6)	
Monthly Household Income (€)		1128.3 (518.6)	

**Table 2:** Professional attributes of the nurses involved in the study (Ν = 115).

		Ν	%
Job position	Support Staff Compulsory Education (YE)	7	6.1
	Nursing Assistant (DE)	88	76.5
	Nurse (TE)	15	13.0
	Nurse PE	5	4.3
Position of responsibility	Yes	13	11.3
	No	102	88.7
Working hours	Morning	20	17.4
	Morning & Afternoon	9	7.8
	Circular	86	74.8
Does/did any member of your family suffer from dementia/Alzheimer’s disease?	Yes	16	13.9
	No	99	86.1
			Median (IQR)
Years of service			5.0 (3–14)
Number of night shifts per month			5 (3–6)
How many weekends do you work on average per month?			2 (2–3)
Number of patients in the department			50.0 (40–50)

**Table 3:** Occupational Settings of the Nurses Involved in the Study (Ν = 115).

		Ν	%
How would you describe the working environment (relationship with colleagues) in your department?	Very poor	2	1.7
	Poor	13	11.3
	Neutral	29	25.2
	Good	49	42.6
	Very good	22	19.1
Are you generally satisfied With the nursing profession?	Not at all satisfied	5	4.3
	Somewhat satisfied	18	15.7
	Quite satisfied	60	52.2
	Very satisfied	23	20.0
	Very much satisfied	9	7.8
What led you to choose the nursing profession? ^1^	My desire to help people	45	39.1
	The increased likelihood of professional rehabilitation	31	27.0
	The influences I received from my environment	31	27.0
	I ended up in this profession by Chance	18	15.7
How do you unwind/decompress from your work? ^1^	Physical rest/sleep	48	41.7
	Physical exercise/gym	31	27.0
	Socializing with friends/family	40	34.8
	Career counseling/supervision	1	0.9
	I do nothing	10	8.7

^1^ Participants could select more than one answer.

**Table 4:** Description of Professional Quality of Life (ProQOL-5) scale.

	Minimum	Maximum	Mean (SD)	Levels		
				Low (≤22)	Moderate (23–41)	High (≥42)
				N (%)	N (%)	N (%)
Compassion satisfaction	20	50	35.4 (7.4)	8 (7.0)	80 (69.6)	27 (23.5)
Burnout	11	41	23.5 (6.4)	53 (46.1)	62 (53.9)	0 (0.0)
Secondary traumatic Stress	10	35	21.5 (5.8)	67 (58.3)	48 (41.7)	0 (0.0)

**Table 5:** Description of HADS scale (N = 115).

	Minimum	Maximum	Median (IQR)
Depression	0	15	4 (2–6)
Anxiety	0	13	3 (1–4)

**Table 6:** Correlations between the ProQOL-5 professional life quality scale and the hospital depression and anxiety scale, via Spearman correlation coefficients (rho) (N = 115).

		Depression	Anxiety
Compassion satisfaction	rho P	−0.37 <0.001	−0.22 0.016
Burnout	rho P	0.44 <0.001	0.44 <0.001
Secondary traumatic Stress	rho P	0.37 <0.001	0.36 <0.001

**Table 7:** Multiple linear regression results with ProQOL-5 as the dependent variable.

Dependent Variable	Independent Variables	Unstandardized Coefficients (Beta)	SE ^+^	95% CI ^++^	Standardized Beta	T	P	Tolerance	VIF
Compassion satisfaction (F = 5.6; *p* < 0.001; R_adj_^2^ = 0.37)	Gender (Females vs. Males)	3.20	1.38	0.47–5.94	0.19	2.32	0.022	0.90	1.12
	Age	0.02	0.07	−0.11–0.15	0.03	0.34	0.733	0.68	1.47
	Married/Cohabiting with partner (yes vs. no)	0.51	1.46	−2.39–3.41	0.04	0.35	0.727	0.58	1.72
	Job position (Nurse vs. Support Staff/Nursing Assistant	3.34	3.25	−3.12–9.8	0.19	1.03	0.308	0.19	5.30
	Circular work schedule (yes vs. no)	−0.94	1.51	−3.94–2.06	−0.06	−0.62	0.535	0.73	1.38
	Number of patients in the department	0.01	0.04	−0.06–0.08	0.02	0.22	0.825	0.89	1.12
	Monthly Household Income	0.003	0.001	0.000–0.006	0.21	2.02	0.046	0.59	1.70
	Working environment (relationship with colleagues) in department ^1^	2.86	0.70	1.47–4.24	0.38	4.09	<0.001	0.72	1.38
	General satisfaction with the nursing profession ^2^	1.89	0.79	0.33–3.46	0.23	2.41	0.018	0.69	1.44
	Educational attainment								
	Vocational Training Institute of Nursing vs. Technical Vocational High School Nursing Department	−1.73	1.37	−4.45–0.98	−0.12	−1.27	0.208	0.73	1.37
	Technological Educational Institute/University/MSc vs. Technical Vocational High School Nursing Department	−4.33	3.11	−10.51–1.85	−0.26	−1.39	0.168	0.18	5.55
	Having a family member with dementia/Alzheimer’s disease (yes vs. no)	−1.59	1.79	−5.15–1.97	−0.08	−0.89	0.377	0.74	1.35
Burnout (F = 5.0; *p* < 0.001; R_adj_^2^ = 0.32)	Gender (Females vs. Males)	0.85	1.22	−1.57–3.28	0.06	0.70	0.486	0.90	1.12
	Age	0.10	0.06	−0.02–0.22	0.17	1.69	0.095	0.68	1.47
	Married/Cohabiting with partner (yes vs. no)	−2.10	1.29	−4.67–0.47	−0.17	−1.62	0.108	0.58	1.72
	Job position (Nurse vs. Support Staff/Nursing Assistant	−0.17	2.88	−5.89–5.56	−0.01	−0.06	0.954	0.19	5.30
	Circular work schedule (yes vs. no)	2.09	1.34	−0.57–4.75	0.15	1.56	0.121	0.73	1.38
	Number of patients in the department	−0.01	0.03	−0.08–0.05	−0.03	−0.38	0.706	0.89	1.12
	Monthly Household Income	−0.001	0.001	−0.004–0.001	−0.10	−0.92	0.359	0.59	1.70
	Working environment (relationship with colleagues) in department ^1^	−2.62	0.62	−3.85–−1.4	−0.41	−4.24	<0.001	0.72	1.38
	General satisfaction with the nursing profession ^2^	−1.39	0.70	−2.78–−0.01	−0.20	−2.00	0.049	0.69	1.44
	Educational attainment								
	Vocational Training Institute of Nursing vs. Technical Vocational High School Nursing Department	0.52	1.21	−1.89–2.93	0.04	0.43	0.669	0.73	1.37
	Technological Educational Institute/University/MSc vs. Technical Vocational High School Nursing Department	0.62	2.76	−4.86–6.11	0.04	0.23	0.821	0.18	5.55
	Having a family member with dementia/Alzheimer’s disease (yes vs. no)	1.34	1.59	−1.82–4.5	0.08	0.84	0.402	0.74	1.35
Secondary post-traumatic stress (F = 2.7; *p* = 0.004; R_adj_^2^ = 0.17)	Gender (Females vs. Males)	1.85	1.26	−0.65–4.34	0.14	1.47	0.146	0.90	1.12
	Age	0.07	0.06	−0.05–0.19	0.13	1.15	0.253	0.68	1.47
	Married/ Cohabiting with partner (yes vs. no)	−1.71	1.34	−4.36–0.95	−0.15	−1.27	0.206	0.58	1.72
	Job position (Nurse vs. Support Staff/Nursing Assistant	2.28	2.98	−3.65–8.21	0.16	0.76	0.448	0.19	5.30
	Circular work schedule (yes vs. no)	0.85	1.38	−1.9–3.6	0.06	0.61	0.541	0.73	1.38
	Number of patients in the department	0.004	0.03	−0.06–0.07	0.01	0.11	0.912	0.89	1.12
	Monthly Household Income	0.002	0.001	−0.001–0.004	0.15	1.29	0.199	0.59	1.70
	Working environment (relationship with colleagues) in department ^1^	−1.76	0.64	−3.04–−0.49	−0.29	−2.76	0.007	0.72	1.38
	General satisfaction with the nursing profession ^2^	−0.42	0.72	−1.85–1.02	−0.06	−0.58	0.563	0.69	1.44
	Educational attainment								
	Vocational Training Institute of Nursing vs. Technical Vocational High School Nursing Department	−3.12	1.26	−5.62–−0.63	−0.26	−2.49	0.015	0.73	1.37
	Technological Educational Institute/University/MSc vs. Technical Vocational High School Nursing Department	−4.07	2.86	−9.75–1.6	−0.30	−1.43	0.157	0.18	5.55
	Having a family member with dementia/Alzheimer’s disease (yes vs. no)	0.26	1.65	−3–3.53	0.02	0.16	0.873	0.74	1.35

^1^ Values could range from 1 (Very bad) to 5 (Very good); ^2^ values could range from 1 (Not at all satisfied) to 5 (Very much satisfied); ^+^ Standard Error; ^++^ 95% Confidence Interval.

**Table 8:** Multiple linear regression with HADS scales as dependent variables.

Dependent Variable	Independent Variables	Unstandardized Coefficients (Beta)	SE	95% CI	Standardized Beta	T	P	Tolerance	VIF
Anxiety (F = 3.4; *p* < 0.001; R_adj_^2^ = 0.24)	Gender (Females vs. Males)	0.083	0.060	−0.036–0.202	0.128	1.39	0.168	0.89	1.12
	Age	0.000	0.003	−0.006–0.006	0.005	0.05	0.960	0.67	1.49
	Married/Cohabiting with partner (yes vs. no)	−0.077	0.064	−0.204–0.050	−0.139	−1.21	0.230	0.57	1.75
	Job position (Nurse vs. Support Staff/Nursing Assistant	0.068	0.141	−0.211–0.347	0.096	0.48	0.631	0.19	5.30
	Circular work schedule (yes vs. no)	0.046	0.066	−0.086–0.177	0.071	0.69	0.491	0.71	1.41
	Number of patients in the department	−0.001	0.002	−0.004–0.002	−0.036	−0.39	0.696	0.89	1.12
	Monthly Household Income	0.001	0.001	−0.001–0.001	0.178	1.42	0.160	0.58	1.71
	Working environment (relationship with colleagues) in department ^1^	−0.069	0.034	−0.137–−0.002	−0.232	−2.04	0.044	0.61	1.64
	General satisfaction with the nursing profession ^2^	0.009	0.035	−0.060–0.078	0.027	0.25	0.801	0.67	1.49
	Educational attainment								
	Vocational Training Institute of Nursing vs. Technical Vocational High School Nursing Department	0.037	0.060	−0.082–0.155	0.063	0.62	0.539	0.72	1.38
	Technological Educational Institute/University/MSc vs. Technical Vocational High School Nursing Department	0.005	0.135	−0.264–0.273	0.007	0.03	0.973	0.18	5.58
	Having a family member with dementia/Alzheimer’s disease (yes vs. no)	−0.071	0.078	−0.226–0.083	−0.093	−0.92	0.362	0.74	1.36
	Burnout	0.024	0.005	0.014–0.035	0.530	4.78	<0.001	0.61	1.64
Depression (F = 4,0; *p* < 0.001; R_adj_^2^ = 0.28)	Gender (Females vs. Males)	0.047	0.052	−0.056–0.150	0.081	0.91	0.368	0.89	1.12
	Age	−0.003	0.003	−0.008–0.002	−0.117	−1.13	0.263	0.67	1.49
	Married/ Cohabiting with partner (yes vs. no)	−0.045	0.056	−0.157–0.067	−0.090	−0.80	0.425	0.57	1.75
	Job position (Nurse vs. Support Staff/Nursing Assistant	−0.053	0.122	−0.296–0.190	−0.084	−0.43	0.667	0.19	5.30
	Circular work schedule (yes vs. no)	0.045	0.058	−0.070–0.159	0.078	0.77	0.441	0.71	1.41
	Number of patients in the department	0.000	0.001	−0.003–0.002	−0.028	−0.31	0.758	0.89	1.12
	Monthly Household Income	0.000	0.0001	−0.001–0.0001	0.063	0.57	0.573	0.58	1.71
	Working environment (relationship with colleagues) in department ^1^	−0.026	0.029	−0.084–0.031	−0.099	−0.91	0.366	0.61	1.64
	General satisfaction with the nursing profession ^2^	−0.017	0.030	−0.077–0.043	−0.057	−0.55	0.582	0.67	1.49
	Educational attainment								
	Vocational Training Institute of Nursing vs. Technical Vocational High School Nursing Department	−0.087	0.052	−0.190–0.016	−0.164	−1.68	0.097	0.72	1.38
	Technological Educational Institute/University/MSc vs. Technical Vocational High School Nursing Department	−0.075	0.117	−0.308–0.158	−0.127	−0.64	0.524	0.18	5.58
	Having a family member with dementia/Alzheimer’s disease (yes vs. no)	−0.059	0.068	−0.193–0.076	−0.085	−0.87	0.388	0.74	1.36
	Burnout	0.016	0.004	0.007–0.025	0.393	3.62	<0.001	0.61	1.64

^1^ Values could range from 1 (Very bad) to 5 (Very good); ^2^ values could range from 1 (Not at all satisfied) to 5 (Very much satisfied).

## Data Availability

The data presented in this study are available upon request from the corresponding author due to privacy and ethical reasons.
